# Resonant metasurfaces at oblique incidence: interplay of order and disorder

**DOI:** 10.1038/srep04484

**Published:** 2014-03-27

**Authors:** M. Albooyeh, S. Kruk, C. Menzel, C. Helgert, M. Kroll, A. Krysinski, M. Decker, D. N. Neshev, T. Pertsch, C. Etrich, C. Rockstuhl, S. A. Tretyakov, C. R. Simovski, Yu. S. Kivshar

**Affiliations:** 1 Department of Radio Science and Engineering, Aalto University, 00076 Aalto, Finland; 2 Nonlinear Physics Centre, Centre for Ultrahigh-bandwidth Devices for Optical Systems (CUDOS), Research School of Physics and Engineering, Australian National University, Canberra ACT 0200, Australia; 3 Institute of Applied Physics, Abbe Center of Photonics, Friedrich-Schiller-Universität Jena, 07743 Jena, Germany; 4 Department of Metrology and Optoelectronics, Gdansk University of Technology, 80-952 Gdansk, Poland; 5 ITMO University, St. Petersburg 197101, Russia; 6 Institute of Condensed Matter Theory and Solid State Optics, Abbe Center of Photonics, Friedrich-Schiller-Universität Jena, 07743 Jena, Germany

## Abstract

Understanding the impact of order and disorder is of fundamental importance to perceive and to appreciate the functionality of modern photonic metasurfaces. Metasurfaces with disordered and amorphous inner arrangements promise to mitigate problems that arise for their counterparts with strictly periodic lattices of elementary unit cells such as, e.g., spatial dispersion, and allows the use of fabrication techniques that are suitable for large scale and cheap fabrication of metasurfaces. In this study, we analytically, numerically and experimentally investigate metasurfaces with different lattice arrangements and uncover the influence of lattice disorder on their electromagnetic properties. The considered metasurfaces are composed of metal-dielectric-metal elements that sustain both electric and magnetic resonances. Emphasis is placed on understanding the effect of the transition of the lattice symmetry from a periodic to an amorphous state and on studying oblique illumination. For this scenario, we develop a powerful analytical model that yields, for the first time, an adequate description of the scattering properties of amorphous metasurfaces, paving the way for their integration into future applications.

The study of metamaterials, i.e. artificial media with unusual electromagnetic properties^1^,^2^,^3^,^4^,^5^, has attracted considerable interest due to many promising applications in a broad frequency spectrum, ranging from radio waves to visible light. Traditionally, metamaterials are realized as periodic arrays of resonant subwavelength scatterers^6^,^7^,^8^. Periodicity is appealing since it often simplifies the design and the simulation of the metamaterial by reducing the consideration of an infinite periodic array to the simpler problem of studying one unit cell only. As a result, periodic arrangements have become commonplace for both bulk metamaterials and their planar analogues called *photonic metasurfaces*, defined as optically dense, planar arrays of resonant scatterers^9^,^10^.

However, there exist two important issues associated with the periodic arrangement of the resonant scatterers to form photonic metasurfaces. First, such lattices exhibit strong spatial dispersion, which was shown to be more pronounced than in random arrays with the same concentration of elements^11^,^12^. Second, perfectly periodic structures are difficult to fabricate, especially if cheap, large scale fabrication is required. State-of-the-art nanolithographic techniques, commonly used to fabricate regular optical metamaterials and metasurfaces, are time-consuming and still comparatively expensive. On the other hand, amorphous metasurfaces can be fabricated faster and cheaper than perfectly periodic structures, since nanometer-resolution lithographic procedures can be replaced by self-assembly methods such as colloidal lithography^13^,^14^,^15^,^16^.

To mitigate the difficulties associated with the periodicity, theoretical studies of amorphous metasurfaces were initiated by several groups^17^,^18^,^19^,^20^,^21^,^22^,^23^,^24^,^25^,^26^,^27^. In particular, it was shown that disorder can substantially modify the optical properties of the lattice of scattering elements^17^,^18^. Of particular interest are amorphous metasurfaces composed of specifically designed scattering elements that sustain both electric and magnetic resonances^19^,^22^,^23^. It was found that the sensitivities of the electric and magnetic resonances to disorder are very different. To understand these differences, an analytical model was recently introduced to analyze periodic, disordered, and amorphous arrays at normal incidence^24^. This model elucidated that in general there are two different loss channels for either resonance, i.e. radiative and absorptive losses. Moreover, one loss channel may clearly dominate over the other at different resonance frequencies of the same photonic metasurface. For example, in the particular configuration considered in Ref. 24, the electric dipolar resonance is dominated by scattering losses, while the magnetic dipolar resonance is dominated by absorptive losses. Furthermore, at the magnetic resonance the electromagnetic field has a much stronger confinement within a single unit cell than at the electric resonance. Therefore, the electric and magnetic resonances respond very differently to an increase of disorder. While the electric resonance is significantly perturbed by disorder, the magnetic resonance remains mostly unaffected.

Although this state-of-the-art model explains the different scattering behavior of multipolar resonances at the transition from periodic to amorphous arrangements at normal incidence, the behavior of these arrays at other angles of incidence remained unclear and so far it is not predictable. However, fully understanding this angular behavior is especially important when considering prospective applications of photonic metasurfaces as spectrally selective surfaces or wavefront shaping devices, which require a carefully tailored optical response at various incident angles. Therefore, the impact of disorder on the scattering properties of optically dense planar arrays of resonant elements illuminated by obliquely incident light requires a dedicated study.

Here we develop a model which predicts the scattering parameters of a very general class of photonic metasurfaces with various degrees of disorder and for any angle of incident illumination. Our model allows the derivation of the effective material parameters - *effective surface polarizability tensors* - and is applicable to both electric and magnetic polarizabilities. This allows us to take into account the response of metasurfaces to both the electric and magnetic components of light. The accuracy of the model is tested experimentally. While for periodic arrangements of scattering elements, the present model neglects the effects of high-order Floquet modes and hence does not predict the corresponding traces in the optical spectra, for amorphous arrangements it suggests an efficient way to properly describe the scattering parameters for any angle of incidence.

The paper is organized as follows. First, the analytical model is introduced for the general case of a photonic metasurface. For a specific example of such metasurfaces, we perform large-scale Finite-Difference Time-Domain (FDTD) simulations at two angles of incident illumination to obtain the corresponding optical responses. From these complex transmission and reflection coefficients we analytically retrieve the collective susceptibility tensors. We then use the obtained susceptibilities to calculate the scattering properties of metasurfaces at any angle of incidence. Next, we probe the predictive power of our model by comparing these results to an experimental study. For this purpose, dedicated nanostructured photonic metasurfaces are fabricated by electron beam lithography. Their optical responses are measured in transmission for a certain range of discrete angles of incidence. We demonstrate good agreement between our analytical predictions and the experimental data.

## Results

First, we introduce our model that allows both transmission and reflection to be found from the known polarizabilities; it also gives the solution of the inverse problem and the definition of the effective polarizabilities of metasurfaces at oblique incidence from the complex transmission and reflection coefficients. A detailed discussion of the model is presented in the *Methods* section of the paper. Here we briefly summarize the final result. We assume that the metasurface is oriented perpendicular to the *z*-axis, and it is placed between two different media with permittivities *ε*^+^ and *ε*^−^. In the common case of omega-type bianisotropy^28^, the polarizability tensors for the metasurfaces can be written in the form: 
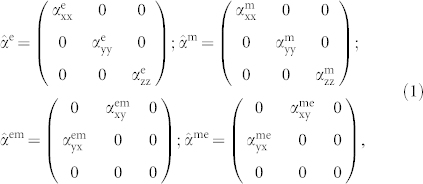
with 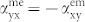 and 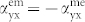, the co polarized reflection and transmission coefficients for the TE and TM polarized incident waves illuminated from *ε*^−^ medium are: 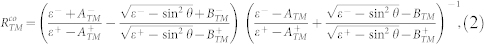

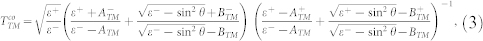

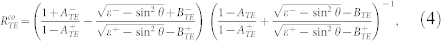

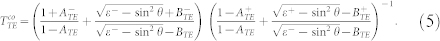
where 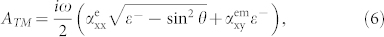

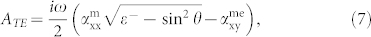

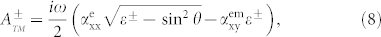

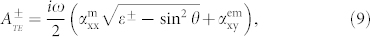

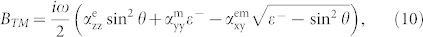

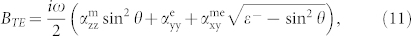

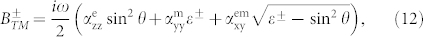

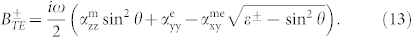


Notice that the cross-polarized components of the reflection and transmission coefficients for this case are zero since there is no cross-components in the electric and magnetic polarizability tensors 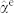 and 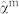 and no co-components in the magneto-electric and electro-magnetic polarizability tensors 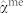 and 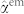.

**Figure 1:**
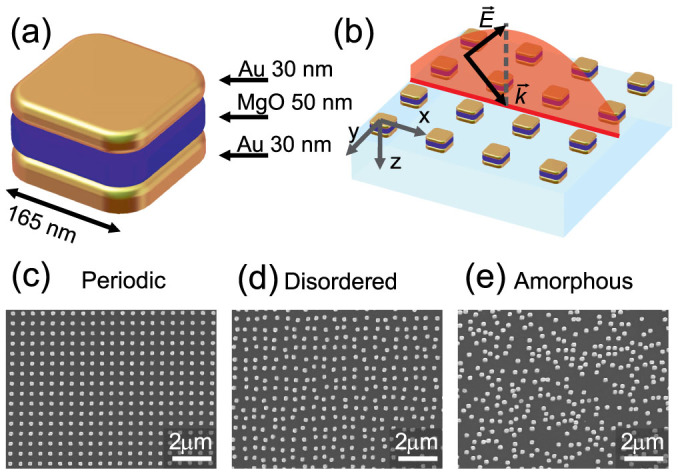
(a) Single nanoparticle, (b) photonic metasurface on a glass substrate under oblique illumination with *p*-polarized light, (c–e) scanning electron microscope images of the fabricated periodic, disordered and amorphous metasurfaces.

Analytical solution of the inverse problem, i.e. finding the polarizability tensors from the known transmission and reflection coefficients, is difficult for this general case due to the large number of tensor components. However, for many practically important cases only a few components are dominant, while the others can be neglected. From here we focus on a particular class of metasurfaces formed by an array of metal-dielectric-metal layered nanoparticles [Fig. 1(a,b)] first proposed in^29^. The use of two metal layers separated by a dielectric spacer allows the response for the *x* and *y* magnetic components of light to be obtained, i.e. nonzero 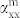 and 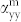, while keeping 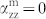. Further, due to symmetry arguments we assume 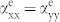, 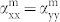. In this case, the two tensors have only three independent components. To retrieve the tensor components we consider normal and oblique illumination of the metasurface by a *p*-polarized (TM polarized) wave with magnetic field in the *y*-direction.

We use the technique described in the *Methods* section to derive the effective polarizabilities 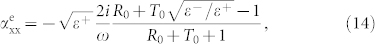

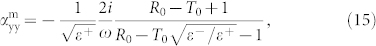

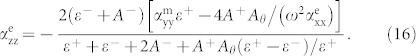
In the Eqs. (14–16), we introduce following shortened notations 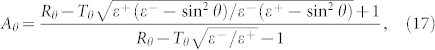

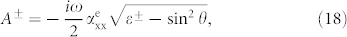

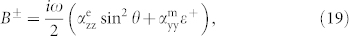
where *θ* is the incident angle in the medium with permittivity *ε*^+^; *R_θ_*, *T_θ_*, *R*_0_ and *T*_0_ are the reflection and transmission coefficients at angle *θ* and at the normal incidence, respectively.

In the following we consider a specific set of photonic metasurfaces which were fabricated (see the *Methods* section). Regarding a single scattering element, we consider a nanoparticle consisting of two gold layers with thicknesses of 30 nm separated by a 50 nm magnesia (MgO) spacer. The size of the nanoparticles at the substrate side is 165 nm × 165 nm. These nanoparticles are assembled in different types of arrangement with a deterministic degree of positional disorder. This disorder is quantified and controlled by the dimensionless disorder parameter *D*, defined as the maximal possible deviation of the position of a nanoparticle in each lateral direction, normalized by the lattice constant^19^. In other words, *D* = 0 corresponds to a periodic lattice arrangement while for *D* = 1 the center position of each nanoparticle can randomly extend between the boundaries of the former unit cell. If *D* is larger than one, each nanoparticle can even be placed beyond these boundaries. For practical reasons, a minimum interparticle separation between adjacent nanoparticles is enforced. We fabricate and study metasurfaces with three different types of arrangements of nanoparticles: (i) a periodic square lattice [*D* = 0, Fig. 1(c)] with a period of 510 nm, (ii) a disordered square lattice [*D* = 0.4, Fig. 1(d)], and (iii) an amorphous arrangement of nanoparticles which can be characterized by an arbitrarily large *D* [Fig. 1(f)]. The average surface density of the nanoparticles is kept identical at 10.5% for all three cases.

**Figure 2:**
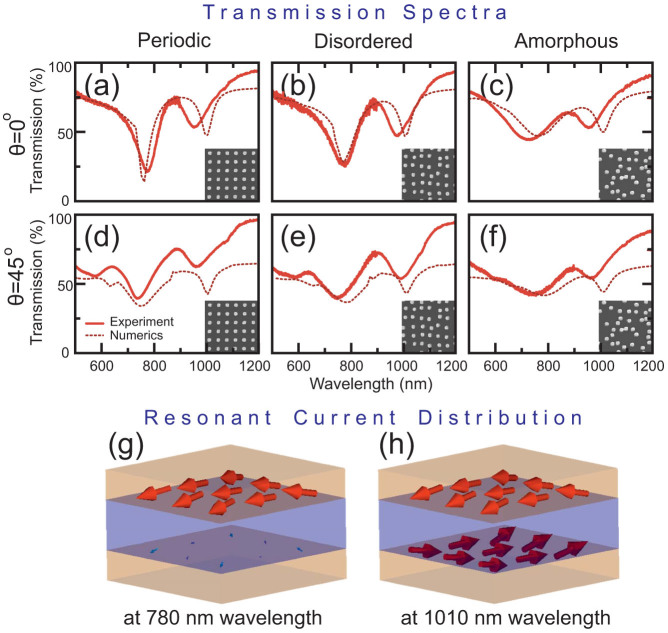
(a–f) Transmission spectra for (a,d) periodic, (b,e) disordered and (c,f) amorphous metamaterials measured experimentally (solid lines) and calculated numerically (dashed lines) for normal (a–c) and oblique *p*-polarized incidence at 45° (d–f). (g,h) Distribution of electric currents in a single nanoparticle at two resonance wavelengths at 780 nm (g) and at 1010 nm (h).

Next we study the optical response of the metasurfaces both experimentally and numerically at normal and oblique incidence [Fig. 1(b)]. See the *Methods* section for details on the numerical calculations. Figure 2 shows the transmission spectra of the metasurfaces under consideration at normal and oblique incidence. All the spectra are dominated by two resonances at characteristic wavelengths around 780 nm and 1010 nm. To assess the type of these resonances, we calculate the distribution of the electric currents in nanoparticles and visualize them in Figs. 2(g,h) with arrows. At the 780 nm resonance, the electric component of the incident wave excites strong electric currents in the upper metal layer and weaker co-propagating currents in the lower layer [Fig. 2(g)]. This corresponds to the excitation of a single electric dipole. At the 1010 nm resonance, counter-propagating currents of comparable amplitude are excited in the two metal layers; clearly indicating the anti-symmetric nature of the mode. This current distribution gives rise to electric quadrupole and magnetic dipole excitations.

**Figure 3:**
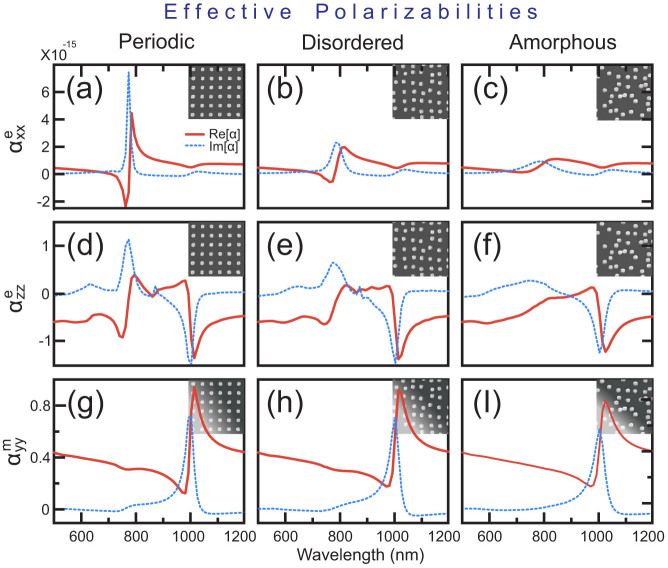
Effective polarizabilities of (a,d,g) periodic, (b,e,h) disordered and (c,f,i) amorphous metasurfaces. Blue, dotted lines correspond to the imaginary parts of polarizabilities; red, solid lines correspond to their real parts.

We then use numerically simulated complex transmission and reflection coefficients to calculate the respective polarizabilities based on Eqs. (14–16). These coefficients were obtained from the simulation under illumination with a plane wave at normal (0°) and *p*-polarized oblique (45°) incidence (see *Methods*). Figure 3 shows the calculated polarizabilities for the three metasurfaces (periodic, disordered, amorphous) under consideration. At the first resonance around 780 nm, we obtain a strong resonant peak of 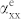, as expected from the electric dipole-type response. At the second resonance around 1010 nm, we observe complex Fano-type^30^ resonant behavior for both 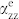 and 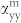, as a result of interference between electric quadrupole and magnetic dipole types of excitation.

**Figure 4:**
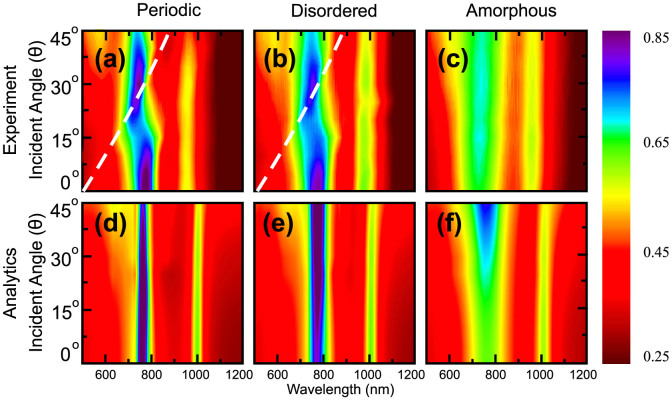
Transmission spectra of (a,d) periodic, (b,e) disordered and (c,f) amorphous metasurfaces, analytically calculated (a–c) and experimentally measured (d–f) for the *p*-polarization of the incident light and the angles of incidence *θ* = 0 … 45°.

By virtue of the Eqs. (2) and (3), we use the retrieved polarizabilities of the three metasurfaces to analytically predict the optical response for any direction of excitation. We verify the applicability of this approach by studying the transmission spectra of the metasurfaces both experimentally and analytically for the case of *p*-polarized illumination and for incident angles between 0° and 45°. Figure 4 shows the corresponding spectra for the periodic [Figs. 4(a,d)], disordered [Figs. 4(b,e)] and amorphous [Figs. 4(c,f)] metasurfaces measured experimentally [Figs. 4(a–c)] and calculated analytically [Figs. 4(d–e)].

## Discussion

As we can observe from Figs. 2–4, both the transmission spectra and extracted polarizabilities demonstrate two very different responses of metasurfaces to the level of disorder. The electric dipole response of the metasurfaces at the 780 nm wavelength is reduced dramatically when the level of disorder is increased. In contrast, the electric quadrupole and magnetic dipole resonant responses at the 1010 nm wavelength remain mostly unaffected by the level of disorder. This is observed in the nearly identical susceptibilities of the metasurfaces at this wavelength.

By analyzing Fig. 4 we find that the analytical results and the experimental data are in good agreement. The main deviation between the theory and experiment is found for periodic and disordered metasurfaces along the marked dashed lines in the experimental spectra. These lines trace Wood's anomaly^31^ are associated with the periodicity of the lattice. The anomaly follows the path, predicted by Rayleigh's formula^32^

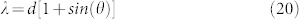
where *λ* is the free-space wavelength and *d* is the lattice period. At these wavelengths an evanescent diffraction order turns into a propagating one that can carry energy into the far-field. This appearance of a new diffraction order is usually accompanied by a sudden redistribution of the diffracted energies in the other orders; causing the sharp features we observe. We point out that the numerical spectra in Fig. 2(d,e) also exhibit a sharp feature at 875 nm wavelength for 45° incidence, which is in excellent agreement with Rayleigh's equation (20). This feature is beyond the predictive power of our model, which assumes the structure to be homogeneous. However, beyond Wood's anomaly our simple model is quite adequate even for the periodic arrangement of the pairs of nanoparticles. Clearly, Wood's anomalies are not observed in the experimental spectrum of amorphous metasurface due to *d* being undetermined. In this case, the surface states are weakly excited and our model predicts adequately the scattering parameters of the array over a broad spectrum of angles of incidence. Finally, we note a strong dependence of the electric dipole resonance mode on the excitation angle, while the second mode consisting of an electric quadrupole and a magnetic dipole remains largely unchanged as the incident angle changes (Fig. 4).

In summary, we have analytically, numerically, and experimentally studied the scattering properties of photonic metasurfaces possessing both electric and magnetic resonant responses with a set of samples allowing access to their transition from periodic to amorphous states. It was confirmed that at oblique incidence positional disorder may have a large impact on the electric dipole response, yet it may have practically no influence on the magnetic dipole and electric quadrupole responses. We have employed an analytical model and concentrated on the scattering of *p*-polarized obliquely incident waves. It was demonstrated that although the model does not take into account Wood's anomalies in periodic arrays, it is adequate and highly effective for the analysis of highly disordered and amorphous arrays. We have shown that our description of metasurfaces in terms of surface polarizabilities is a powerful tool applicable to the characterization of a very general class of photonic metasurfaces with disordered and amorphous states. Our model may simplify considerably the optimization of prospective metasurfaces. The theory established herein provides a previously missing link for the analytical treatment of a newly emerging class of photonic metasurfaces fabricated by innovative self-assembly methods and for the first time allows the prediction of their spectrally and angularly resolved optical properties.

## Methods

### Analytical model

Let us consider a planar array of resonant nanoparticles located in the *xy*-plane, centered at *z* = 0 [Fig. 1(b)] between two media with the permittivities *ε*^+^ and *ε*^−^. The distances between the neighboring particles are assumed to be smaller than the wavelength of the incident electromagnetic field. We then consider the array as a thin effective medium, possessing two surface polarization densities: electric **P** and magnetic **M** respectively. We assume excitation of the array with a plane wave from the side of the *ε*^+^ medium.

First, we neglect the evanescent waves at the interfaces and write relations between the surface polarization densities and the averaged electric **E_av_** and magnetic **H_av_** fields: 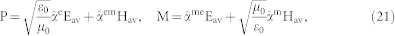
where 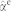, 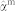, 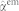, and 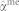 are the electric, magnetic, electro-magnetic, and magneto-electric effective polarizability tensors and 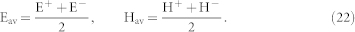
are the averages of the fields at the top **E**^+^ and at the bottom **E**^−^ of the arrays around *z* = 0.

Second, we write Maxwell's boundary conditions at the interface^33^: 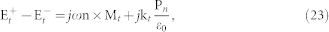

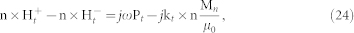

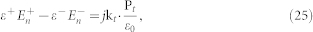

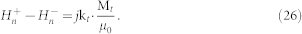
where **n** is the unit vector perpendicular to the array plane and the indices *_t_* and *_n_* correspond to tangential and normal components of the fields and polarization densities, and **k***_t_* is the tangential component of the propagation vector **k**.

Third, we employ the relations between the magnetic and electric fields **H**, **E** and the dyadic transmission and reflection (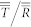) coefficient. If we assume plane wave illumination from the side of the *ε*^+^ medium which is in the *z* > 0 half space then the incident (**E**^inc^), reflected (**E**^ref^), and transmitted (**E**^tr^) electric fields are: 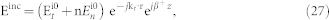

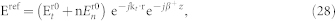

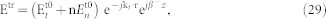
respectively. The tangential components of the magnetic and electric fields correspondingly have the following relations: 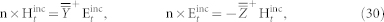

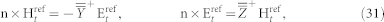

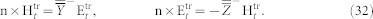
Where: 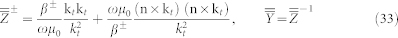
Note that **k***_t_***k***_t_* and (**n** × **k***_t_*) (**n** × **k***_t_*) are two orthogonal dyads. There are also the following relations between the normal components of the electric/magnetic field and the tangential components of the magnetic/electric field which are needed in the our calculations; i.e.: 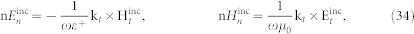

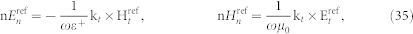

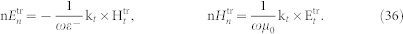
In equations (27–29) we have denoted 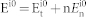, 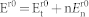, and 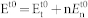 as the amplitude of incident, reflected, and transmitted fields respectively, **k**^±^ = **k***_t_* + **n***β*^±^ (with 
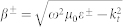
) as the propagation vector in medium *ε*^±^ and 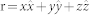 as the position vector. Now equations (23–26) may be solved to find the relations between 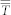, 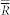 and 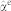, 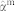, 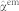, 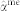 using (27–36) and: 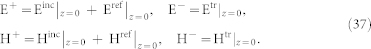
Note that the general definitions for the dyadic transmission and reflection coefficients are: 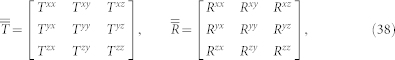
where 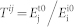 and 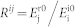 with *i*, *j* = *x*, *y*, *z* (in the Cartesian coordinate system).

Equations (21)–(38) show the general algorithm to find the vectorial form of the reflection and transmission coefficients. However, in practice, we are most interested in transverse magnetic (TM or p) or transverse electric (TE or s) polarized waves. In these cases we need to decompose equations (21)–(38) for each case and to solve the problem for the reflection and transmission coefficients separately. For example, based on the incident wave polarization one can find the cross and co-components of the reflection for the TM and TE cases in terms of the tangential components of the 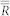 tensor; i.e., 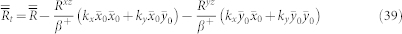
as: 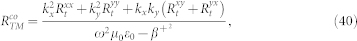

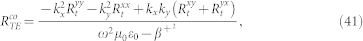

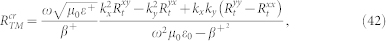

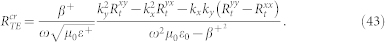


The same expressions hold for the transmission coefficients by changing the + signs to − signs for *ε* and *β*.

We point out that solving equations (21)–(38) for the most general case is challenging due to the large number of components of the tensors. However, for many cases a substantially smaller number of independent non-zero components of the tensors can be taken into account. Analytical solution for one practically important case is presented in the *Results* section.

### Numerical calculations

The majority of numerical calculations were performed using an in-house FDTD method^34^,^35^. The transmission and reflection for the periodic metasurface were calculated using a single unit cell with periodic boundary conditions parallel to the metasurface and perfectly matched layers normal to the metasurface. For the simulation of the disordered and amorphous arrangements we similarly employed a super-cell approach. Each periodic super-cell contained 225 particles and had a total size of 7.65 × 7.65 *μm*^2^. For each wavelength we used monochromatic plane wave excitation to properly take into account the dispersion of the metal.

In addition, numerical calculations were performed with CST Microwave Studio to visualize the distribution of the electric currents at resonant wavelengths.

### Experiments

The samples were fabricated using electron-beam lithography in order to precisely control the nanostructure positions and hence the deterministically induced disorder. After patterning the resist with the variable beam shape electron-beam writer Vistec SB350 OS, the nanoparticles were obtained by the thermal evaporation of a sequential gold/magnesia/gold layer stack and a subsequent lift-off procedure.

The transmission spectra for normal incidence were double-checked as described in Ref. 19. Angle-resolved measurements were performed within the wavelength range from 500 nm to 1200 nm and in steps of 5° from *θ* = 0° up to *θ* = 45° with respect to the optical axis. These spectra were measured with a home-made white-light spectrometer setup consisting of a tungsten light source and an optical spectrum analyzer. In all measurements, the incident light was *p*-polarized and the azimuthal angle was kept constant at *φ* = 0° (aligned along one of the principal axes of the reference periodic array). The same orientation between the sample and the azimuthal angle was kept for the disordered and amorphous metasurfaces.

## Author Contributions

M.A., S.T., T.P. and C.S. conceived the original idea which was further developed by S.K., D.N. and Y.K. S.K., C.H., D.N., T.P. and C.R. designed the experiment. C.H. fabricated the sample. M.A. developed the analytical model. Experimental data was generated and double-checked at different facilities by S.K., C.H., M.K., A.K. and M.D. Numerical simulations were carried out by M.A., S.K., C.M. and C.E. Theoretical considerations were contributed by all authors. S.K. and M.A. wrote the initial version of the manuscript. All the authors have analyzed and discussed the results thoroughly and contributed significantly to writing of the manuscript.
